# Mapping urban greenspace use from mobile phone GPS data

**DOI:** 10.1371/journal.pone.0248622

**Published:** 2021-07-07

**Authors:** Meghann Mears, Paul Brindley, Paul Barrows, Miles Richardson, Ravi Maheswaran

**Affiliations:** 1 Department of Landscape Architecture, University of Sheffield, Sheffield, South Yorkshire, United Kingdom; 2 Human Sciences Research Centre, University of Derby, Derby, Derbyshire, United Kingdom; 3 Public Health GIS Unit, School of Health and Related Research, University of Sheffield, Sheffield, South Yorkshire, United Kingdom; Bruno Kessler Foundation, ITALY

## Abstract

Urban greenspace is a valuable component of the urban form that has the potential to improve the health and well-being of residents. Most quantitative studies of relationships between health and greenspace to date have investigated associations only with what greenspace exists in the local environment (i.e. provision of greenspace), rather than to what extent it is used. This is due to the difficulty of obtaining usage data in large amounts. In recent years, GPS functionality integrated into mobile phones has provided a potential solution to this problem by making it possible to track which parts of the environment people experience in their day-to-day lives. In this paper, we demonstrate a method to derive cleaned, trip-level information from raw GPS data collected by a mobile phone app, then use this data to investigate the characteristics of trips to urban greenspace by residents of the city of Sheffield, UK. We find that local users of the app spend an average of an hour per week visiting greenspaces, including around seven trips per week and covering a total distance of just over 2.5 km. This may be enough to provide health benefits, but is insufficient to provide maximal benefits. Trip characteristics vary with user demographics: ethnic minority users and users from more socioeconomically deprived areas tend to make shorter trips than White users and those from less deprived areas, while users aged 34 years and over make longer trips than younger users. Women, on average, make more frequent trips than men, as do those who spent more time outside as a child. Our results suggest that most day-to-day greenspace visits are incidental, i.e. travelling through rather than to greenspace, and highlight the importance of including social and cultural factors when investigating who uses and who benefits from urban greenspace.

## Introduction

There is substantial evidence that urban greenspace can improve the health and well-being of residents for a range of outcomes, including lowering all-cause mortality, improving general and cardiovascular health, increasing birth weight, reducing overweight/obesity and cardiovascular disease, and reducing rates of mental health disorders [1–6]. Moreover, greenspace has the potential to reduce the health inequalities associated with socioeconomic deprivation [6–9].

The health benefits of urban greenspace likely arise through the interaction of multiple pathways. Greenspaces are able to mitigate harms to health that are caused by the urban environment, by providing respite from air and noise pollution associated with traffic and industry, and locally mitigating the urban heat island effect through shade provision and evapotranspirative cooling [2,3]. Spending time in more natural environments can improve well-being through affect regulation and also restore psychological capacities, through reducing stress and thereby increasing positive emotions; by facilitating recovery from attentional fatigue; and/or through an evolved psychological reward system for strong connections with nature [3,10–14]. Experimental studies simulating exposure to greenspace in a controlled environment demonstrate these psychological benefits through both subjective measures, i.e. self-report, and objective measures such as electroencephalogram and blood pressure [3,15–19]. The benefits of greenspace are not necessarily constrained to being in the greenspace but may also affect surrounding areas–such is the case for greenspace in reducing the urban heat effect for wider geographic areas but also in the visual connection beyond the greenspace itself. It is also possible that urban greenspaces provide suitable environments for the promotion of both social contacts and physical activity; although the evidence for these pathways is less conclusive [2,3,20].

Urban greenspace therefore has potential as means of improving population health, with a few preliminary analyses generally indicating a good level of cost-effectiveness [1,21]. However, a major limitation of the vast majority of epidemiological studies of relationships between health and greenspace is that only a broad measure of the presence (provision) of greenspace near to houses is used [3]. These measures are usually derived from either a vegetation index (e.g. Normalised Difference Vegetation Index) indicating the presence of photosynthetically active plants, or from GIS land cover/land use data showing areas of natural land covers [3,5]. Such measures give no indication of greenspace *use*, which likely provides the majority of health benefits [3,5,20].

Data on how people use and experience greenspaces are not generally available. This is of considerable importance, given that the existence of greenspace in the local environment will not provide equal benefit to all residents: the uses and meanings of greenspace depend on a range of demographic and cultural factors [22–24]. For example, older people and people with poor health are usually found to be less likely to use greenspace, as are those affected by time or financial constraints, which disproportionately affect women [24–26]. A feeling of social exclusion, and perceived lack of safety are also important factors [23–25]. Ethnicity and cultural heritage are important factors determining greenspace preferences and uses [24,27], with close relationships with nature interacting with visits to green spaces to increase wellbeing outcomes [14]. Ethnicity may lead to distinct perceptions and patterns of use in urban green space, and affects motivational reasons for use [28] with research in the US suggesting that some minority ethnic groups are more likely to prefer recreation to conservation [27] and more constrained by time as a reason for not visiting parks [24]. Research in England suggests that ethnic minority groups may have visited natural environments less frequently [29] and were less likely to use parks for exercise [30]. Reasons for differences are complex and Weber and Sultana [31] hypothesised that socioeconomic marginality, differing cultural norms, and the lingering legacy of discrimination were all important.

These issues relate more generally to the Unknown Geographic Context Problem (UGCoP), whereby the ability to understand how geographic context affects behaviours and outcomes is hampered by uncertainty about how individuals experience their environment on a day-to-day basis [32]. GPS-enabled mobile devices show promise as a means of overcoming the UGCoP, by facilitating the collection of data on how people behave in both spatial and temporal dimensions, and thereby enabling an improved understanding of people’s exposure to relevant environments [32–35].

Data from GPS-enabled mobile devices has revolutionised travel surveys [36–38], and its promise in health studies, particularly studies of physical activity and exposure to hazards, has also been recognised [34,39–41]. The use of GPS data is adding to diary-based studies and providing more accurate specifications of both activities and environmental exposures [34,35,37]. GPS has also been used to study the behaviour of visitors to particular greenspaces [42,43]. GPS data can be used to analyse where people come from, and where they go once within a greenspace.

A drawback of GPS data, however, is that the datasets are frequently large and challenging to clean and interpret [36,38]. Two main approaches to cleaning and interpreting GPS data, i.e. identifying relevant periods of activity and minimising the influence of errors, exist in the literature: machine learning approaches and procedural approaches [44]. Machine learning approaches use a variety of automated computational algorithms (sometimes in combination with pre-defined rules) to attempt to discriminate, for example, the ends of trips and different modes of transport [38,41,44–48]. Procedural approaches, on the other hand, use only rulesets based on assumptions about behaviour [39,44].

A key challenge in either approach is to account for errors inherent in GPS data. GPS devices take time to obtain an initial position fix after being turned on or emerging from an area without satellite reception [39,49]. This can result in missing data at the start of trips. GPS accuracy is also an issue in urban environments in particular, because buildings and tree canopy cover can cause either complete signal loss, or signal scatter resulting in incorrect positioning [39,40,49,50]. Studies of mobile device GPS accuracy in urban environments have found average horizontal accuracies ranging between around 5 to 20 m, depending on the device used as well as environmental conditions [50]. While dedicated GPS devices can be more reliable than GPS integrated into mobile phones, using dedicated devices adds considerable costs to studies [50–52]. Although not due to error, data may also be missing from the end of trips due to battery depletion resulting in GPS or the mobile device being switched off [40].

Our focus on greenspace raises specific issues related to positional accuracy, for example consideration of entrance points used to enter and exit greenspaces, and whether a user might be walking along a path on the outside of a greenspace boundary. Other studies [37,53] tend to remove potential data errors via procedures that consider only the GPS data but not on-the-ground geographic features, such as removing data with excessive speeds. The data cleaning adopted here undertakes a more comprehensive approach, encapsulating uncertainty within the underlying data through the use of additional GIS data.

In this paper, we use a procedural approach to infer trip-level information from GPS data collected by the Shmapped mobile device app. Shmapped was used to deliver a quasi-experimental well-being intervention that prompted users to notice nature when visiting urban greenspaces [54,55]. Full details of the intervention and its outcomes can be found in McEwan et al. [54]. Consent was also obtained from users for GPS tracking during time spent within greenspaces [55].

We use this data to analyse the characteristics of trips to greenspaces: (1) how long users spend in greenspaces; (2) how far they travel within them; (3) how far from home they travel to visit them; (4) average speeds of users; and (5) types of greenspaces visited. We then investigate whether trip-level characteristics are associated with selected demographic characteristics (age, gender, ethnicity and socioeconomic deprivation). We also compare trip-level characteristics to those from two datasets curated using more traditional survey methods: a survey by the local council into residents’ greenspace use, and perceptions and problems associated with greenspaces; and Monitor of Engagement with the Natural Environment (MENE), a multi-year, England-wide survey of greenspace visitation habits and attitudes.

To our knowledge, this is the first paper using GPS data to specifically investigate adults’ greenspace-visiting behaviour (although Olsen et al. [35] and Wheeler et al. [53] investigate children’s activity in urban areas, including to greenspaces). After describing a method for cleaning and post-processing the GPS data, we show the importance of attending to the details of post-processing by illustrating differences between minimally and fully processed and cleaned data.

## Methods

### GPS data

Respondents were drawn from users of the Shmapped app. Promotion of the app was through a variety of mechanisms including social media; distributing posters and leaflets; through conservation organisations (namely the Wildlife Trusts), Council staff, large local employers, and General Practitioners (GPs). Comprehensive details of the recruitment strategy can be found in associated publications [54,55]. The Shmapped app collected GPS location data whenever users entered or travelled close to urban greenspaces. The app was developed as part of the Improving Well-being through Urban Nature project (see project website at http://www.iwun.uk for full details), which investigated how urban greenspace and other urban nature in the city of Sheffield, UK, can improve residents’ health and well-being. Consequently, although users from anywhere could download Shmapped, GPS data were only collected for visits to greenspaces in Sheffield. The study was approved by the Human Sciences Research Ethics Committee at the University of Derby (Ethics Ref No: 08-1617-KMp). Demographic characteristics of Shmapped users recording at least one visit to greenspace are shown in Table 1. Users self-identified the demographic data provided through Shmapped (age, gender, ethnicity). Deprivation was assigned using the UK Index of Multiple Deprivation (IMD 2015) using the home postcode location provided by the user when installing Shmapped. IMD is available as deciles—calculated by ranking all areas in England from most deprived to least deprived and dividing them into 10 equal groups. The categorises used for Ethnicity are those found within the UK 2011 Census–which allows exploration of the representativeness of the sample. The distribution of user socioeconomic details is shown in comparison with that of Sheffield’s wider population in S1 Fig.

**Table 1:** Demographics of Shmapped users recording at least one valid trip.

Characteristic	Statistic	Value
Sample size	Total n	888
	Recording 1+ valid trips	577
	Recording 1+ trips with 0 flags	561
Age	Range	18–71
	Mean	33.1
	SD	12.5
	n	530
Gender	Male	348 (66%)
	Female	182 (34%)
	Other	1 (<1%)
	n	531
Ethnicity	Asian or British Asian	48 (9%)
	Black or Black British	4 (<1%)
	Mixed	14 (3%)
	White	443 (83%)
	Other	19 (4%)
	n	531
IMD decile	Range	1–10
	Mean	6.0
	SD	2.8
	n	534

Geofences circumscribing the areas within 10 m of a greenspace were used to trigger the app to collect GPS data. Due to mobile device operating system limitations, geofences were circular, but in order to preserve user privacy data points recorded more than 10 m from boundaries were not sent to the server for storage. Greenspace boundaries were provided by Sheffield City Council and compromise the 945 green and open spaces identified in the council’s 2007 assessment of outdoor recreation and leisure provision [56]. A more detailed description of the distribution of greenspace within Sheffield can be found in associated publications [57,58]. For a full description of the app and its effectiveness as an intervention, see McEwan et al. [54,55]. Collecting data when users were within 10 m of greenspace boundaries was implemented to allow for the typical horizontal positional error found in the types of GPS receivers found in civilian mobile devices [50].

In total, 656,000 GPS data points were collected from 888 mobile devices, in the period from 1^st^ July 2017 until 6^th^ October 2018. GPS data collection process was automatic, with no user input or prompting after consent had been provided within the app. Data points were not split into individual “trips” in the raw data, and did not otherwise have any semantic data attached.

### Data processing

**Fig 1:**
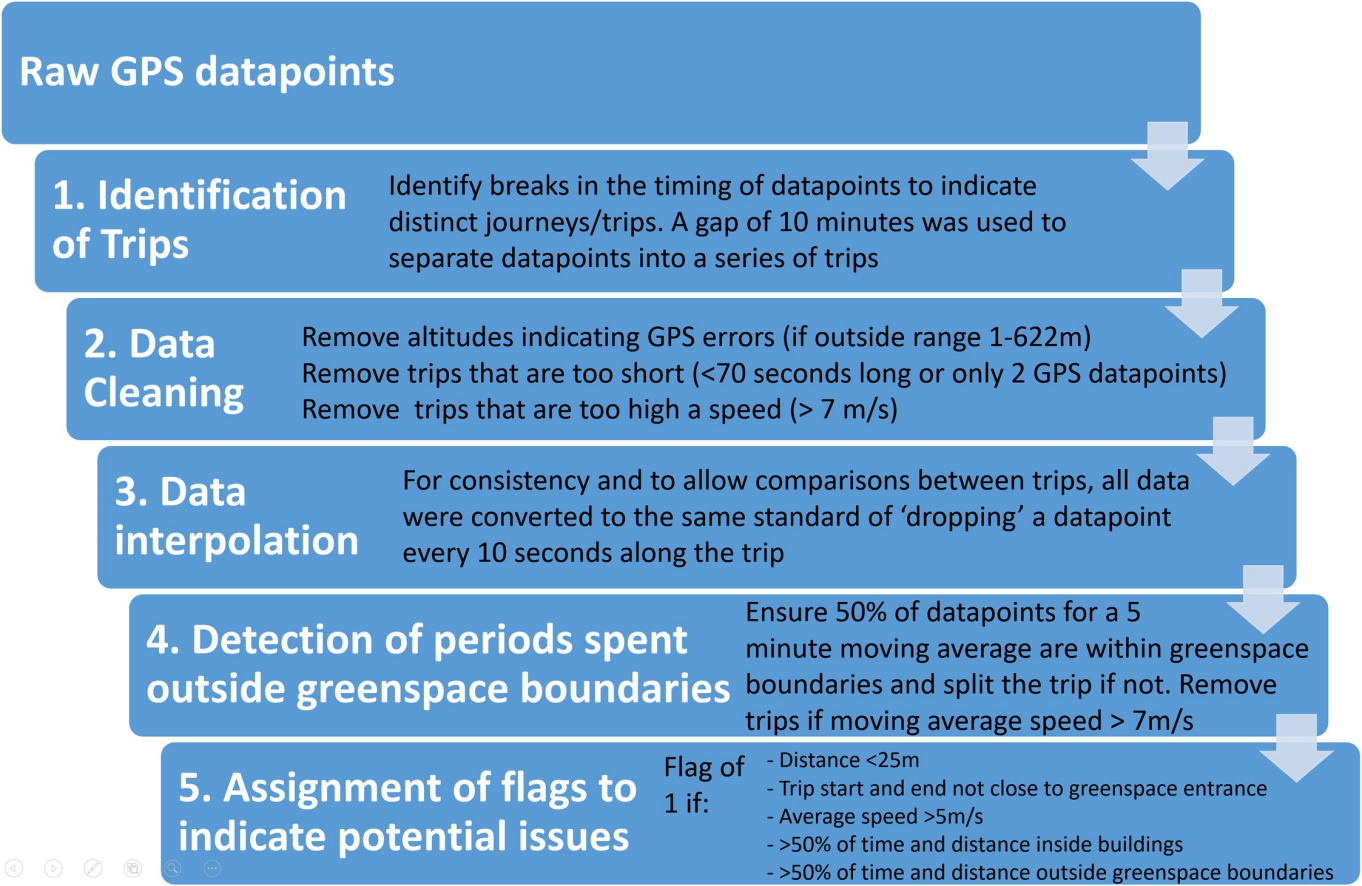
Flowchart summarising the data processing stages.

The process of extracting trip-level data from the raw GPS data points comprised several stages–as outlined in Fig 1. First, GPS points were divided into trips, then cleaned. Next, interpolation was used to obtain polylines with vertices corresponding to regular time intervals. Interpolation was performed because vertices representing equal time intervals were found to be helpful for further post-processing. The final stages of post-processing involved cropping the starts and ends of journeys to greenspaces, and checking the validity of trips as representations of single, non-vehicular visits to greenspace. Full details are given in the following sections.

All data processing was performed in R [59]. The Tidyverse packages [60], and the package ‘sf’ [61] for spatial processing, are central to analysis. The packages ‘data.table’ [62], ‘lubridate’ [63] and ‘smoothr’ [64] are also used. A generalised version of the R script is available at https://github.com/MeghannMears/GreenspaceGPS.

#### Data requirements

We used the following attributes from GPS data: geographic coordinates, including altitude; unique device identifier; and a timestamp. The app requested GPS location every 10 seconds. Three additional GIS datasets were used during processing: boundaries of greenspace (polygon data); greenspace entrance points (point data); and locations of buildings within greenspaces (polygon data). To calculate the distance between users’ home locations and the start of trips, home location data at unit postcode level (point data) was used.

Greenspace access points were derived from a combination of data sources including Sheffield City Council Parks and Countryside data; Ordnance Survey (OS) Open Greenspace; OpenStreetMap; OS Integrated Transport Network; OS MasterMap topography layer; aerial imagery; Google StreetView and site visits. For full details of this dataset, see Mears et al. [57]. Buildings within greenspaces were identified from OS MasterMap topography layer.

#### Stage 1: Identify ‘trips’

As the dataset was not divided into individual trips when a user entered and later left a geofenced area, the first step of processing was to identify such breaks. This was achieved by identifying time gaps of an appropriate length (for example where there was a gap of greater than x minutes between one GPS recording and the next). Although we were unable to find previous studies that have used time gaps to identify trips, studies that have identified stops *within* trips have typically used periods of two to five minutes [36,38,45]. However, Schuessler and Axhausen [49] noted that longer periods may be appropriate in the case of poor signal reception, and indeed we found a period of ten minutes to be more appropriate here.

#### Stage 2: Data cleaning

Following Schuessler and Axhausen [49], as the first step in data cleaning we removed data points that were outside of the altitudinal range of Sheffield (19–592 m above sea level) as these frequently will refer to data errors from incorrectly received satellite signals. We allowed for a 30 m error buffer on the upper limit [38], but due to a high number of points erroneously reporting an altitude of +1 to -1 metres, we set the lower error buffer to 1 m.

We also removed trips that were so short as to suggest that either a substantial amount of data was missing, or it was more likely that a user was passing by a greenspace (within the 10 m geofence) than actually entering it. Trips lasting less than 70 seconds [37] or containing only one or two GPS points were removed.

The final step of data cleaning involved consideration of speed. We used the approach described by Schuessler and Axhausen [49], who identified jumps in position where the user appears to move faster than is reasonably likely, e.g. due to urban canyon-related GPS errors [49]. In applying the method to our study, we used a threshold speed that additionally aimed to remove trips where users are travelling by vehicle, most likely on the outside edge of a greenspace (but within the 10 m buffer). The movement speed (calculated as 3D Euclidean distance) between each consecutive pair of points was calculated, and the trip was split into segments where the speed was below the limit. These segments are termed ‘quality segments’. Each pair of consecutive quality segments were then compared, and then shorter was removed. This process was repeated until the entire trip had a speed under the determined limit. Studies using GPS-reported movement speed to identify transport mode find that pedestrians, runners and cyclists rarely move faster than 7 metres per second (m/s which equals 25 km/h) [41,44,46–48,65]. While cyclists may sometimes move faster than this, faster speeds overlap with the average speed range of buses and cars in urban areas, so we set the speed limit at 7 m/s, plus 20 m to allow for GPS error [49]. Finally, trips that no longer had GPS points for a duration of at least 70 seconds were removed.

#### Stage 3: Data interpolation

The temporal density of the GPS data points varies considerably, due to loss of signal as well as removal of erroneous data points during cleaning. Prior to further processing, it was therefore useful to create spatial points with approximately equal time intervals. This also facilitated calculation of derived trip attributes (e.g. time spent in/outside of park boundaries) and made visual interpretation of speed possible via vertex density. We used linear interpolation to create interpolated GPS paths, with a vertex located approximately every 10 seconds of travel. 10 seconds is a common recording interval used for GPS data and aligns with the app requesting GPS location every 10 seconds.

We explored the possibility of smoothing the data at this point, to reduce data artefacts caused by random error [49]. However, we found that smoothing even with a small bandwidth caused GPS traces that appeared in reality to follow paths near to greenspaces to intersect with greenspace boundaries. Furthermore, we would expect people in greenspaces sometimes to take meandering routes. We therefore did not perform data smoothing.

#### Stage 4: Detection of periods spent outside greenspace boundaries

Using the interpolated trip path, it was possible to detect trips that included extended periods of time spent outside of greenspace boundaries or spent travelling at higher speeds than expected for non-vehicular travel. Checking for this was necessary because, due to the collection of GPS data when users were within 10 m of boundaries, many apparent trips actually show users travelling along the outside of greenspace boundaries for part or all of the trip. Due to positional error in GPS data [49,50] that may cause data points recorded just inside boundaries to report a position just outside, or to report a high speed over a short period, we calculated a moving average of the proportion of vertices inside boundaries over approximately five minutes of travel. If at any point the moving average dropped below 50% inside boundaries, the interpolated path points <50% were discarded.

We were also able to undertake a more fine-grained analysis of speed, with a lower allowance for error than was undertaken during data cleaning (stage 2), to identify periods within trips when average speeds were greater than is likely for non-vehicular travel. Again using a moving average over five minutes, points with a moving average speed greater than 7 m/s were discarded.

If points were discarded from the middle of a trip, the trip was split into two (or more).

If the total trip length was less than five minutes, then rather than using a moving average, the proportion of vertices inside boundaries and average speed were calculated for the entire trip. If the proportion was less than 50% or speed was greater than 7 m/s, the entire trip was discarded; otherwise, no changes are made.

Following this process, in order to remove shorter periods at the start and end of trips that occurred outside boundaries, points were discarded so that there was only one vertex outside of boundaries at the start and end of trips.

#### Stage 5: Trip analysis

We calculated basic trip attributes including trip length, duration, average speed, and distance from home postcode to start of trip. Finally, we calculated additional measures that can be used to determine how likely trips are to be valid representations of a pedestrian trip to a greenspace as well as to analyse trips. These include:

- The length of the interpolated path that is within greenspace boundaries; and the number of unique greenspaces visited.
- The distance of the interpolated trip start and end points to greenspace entrance points by using a nearest neighbour search. If the trip did not start and finish close to a greenspace entrance, it is not clear that the greenspace was entered. Note that this depends on having complete greenspace entrance data, which is challenging where informal entrances may be made (e.g. broken fences).
- The percentage of the trip (in terms of both distance and time) that occurs inside buildings. If a large proportion of the trip is spent inside buildings, it is likely that the user was visiting the building rather than the greenspace.

Following calculation of these measures, we applied several criteria to determine the level of certainty that the trip data represents a genuine and complete non-vehicular visit to a greenspace. These criteria are described in Table 2. Hereafter, these criteria are referred to as ‘flags’, and highlight the following potential issues with trip data: short distance; incomplete data; high speed; time spent inside buildings; and time spent outside greenspaces. In essence, these levels of certainty are similar to sensitivity analysis in that they allow interpretation of the extent to which trips data are dependent upon the criteria.

**Table 2:** Criteria for determining level of certainty in whether trip data is an accurate representation of a non-vehicular trip to a greenspace.

Criterion	Rationale
Distance > 25 m	A trip during which the GPS fails to report movement may reflect failure of the GPS receiver to update e.g. due to signal blocking [49]–though it may also indicate that the user is stationary while the GPS is active. We do not consider very short journeys to comprise a meaningful trip to greenspace.
Trip start and end < = 25 m from a greenspace entrance point	If no passage near to an entrance point is captured, it is probable that part of the trip data is missing–although it is also possible that the entrance point dataset is incomplete.
Average speed < 5 m/s	While we have used 7 m/s (25 km/h) as the upper acceptable speed elsewhere to ensure that all non-vehicular travel is captured, from previous studies we consider that 5 m/s (18 km/h) will capture the vast majority pedestrians, runners, and cyclists [41,47,48], as well as mobility scooter users. This may help to remove some journeys undertaken by vehicle at low speeds (as might be expected in pedestrianised areas such as parks).
> = 50% of time and distance spent outside of buildings	If more than half of the journey is spent inside buildings inside greenspaces, we consider the purpose of the trip to be to visit the building–although this does not preclude receiving benefits from passing through the greenspace.
> = 50% of time and distance spent inside greenspace	If more than half of the journey is spent outside of greenspaces, we consider it unlikely that we have correctly captured a visit to greenspace. (Note that the moving average removal of trips going outside of greenspace boundaries only considered time, not distance.)

### Summarising trip characteristics

After completing data processing, we summarised the following characteristics of trips using the mean, standard deviation and median, as well as visualising the distribution using histograms: trip duration, distance covered, distance spent in greenspaces, average speed, and distance from user’s home to start of trip. Each of these were calculated for the raw data (i.e. following initial splitting by time gaps, with no cleaning or post-processing); for cleaned data; and for fully post-processed data, both including all trips (regardless of number of flags) and only trips with zero flags. These varying sets of data represent increasing confidence in the output through removal of probable data errors.

### Summarising user-averaged trip characteristics

In addition to summarising trip characteristics across all trips, we also created summaries using data averaged first to user-level, such that each user contributed equally to the summary values, rather than each trip contributing equally. These are hereafter referred to as user-averaged trip characteristics. This was performed in order to facilitate comparisons with survey data, which also have a single data point per user, and to ensure that individuals with high numbers of greenspace visits did not skew interpretations.

Additionally for users, we calculated the frequency of greenspace visits across the period that the user was recording trips. This was used to calculate the average amount of time that the user spends in greenspace over longer periods of time. These statistics were calculated for all users and only for those reporting at least five trips; and for all trips and only those with zero flags.

#### Comparison with Monitor of Engagement with the Natural Environment survey data

The MENE survey is carried out by Natural England, the governmental agency responsible for protecting England’s natural environment, as a means of tracking use of and attitudes towards the natural environment [66]. The survey has been carried out on an on-going basis since 2009. During the MENE interview, respondents are asked about a randomly selected trip to greenspace from the past 7 days. Amongst the data collected about this trip is the trip duration, and distance travelled to the greenspace. MENE data for the period 2009–2019 were downloaded from the Natural England website (http://publications.naturalengland.org.uk/publication/2248731, date accessed 19/12/2019) and used to obtain the distributions of these variables. We excluded visits that were not to greenspaces in a town or city, in order to exclude visits to types of greenspace e.g. countryside or coastal spaces that were not included amongst Shmapped’s geofences. These data were compared with the user-averaged distributions from the Shmapped data.

#### Comparison with Sheffield City Council Parks and Countryside survey

Through partnership with Sheffield City Council, we obtained the results of the Parks and Countryside department’s customer satisfaction survey for 2019. In this survey, residents are asked about the park or greenspace that they visit most frequently. One of the questions asks how far this greenspace is from their home address. These data were compared with user-averaged distributions from Shmapped data.

### Summarising trip destinations

We summarise trip destinations first by type of greenspace, as classified in Sheffield City Council’s 2007 audit of green and open spaces (the data source used to geofence greenspaces). This was undertaken using overall (not user-averaged) data: each trip was counted individually, and if a trip included greenspaces of multiple types, both were counted. This facilitated comparison of trip destinations with the distribution of types of greenspace in Sheffield. We also undertook limited comparison of the distribution with data from MENE regarding the type of greenspace visited in the randomly selected visit, although due to incompatible typologies it was not possible to compare all categories.

Second, we used the number of users visiting specific named greenspaces as validation of our data processing approach, using the Sheffield City Council survey. This survey asked respondents which greenspace they visited most. For this analysis, we used user-averaged data, i.e. each user was only counted as visiting any individual greenspace once, regardless of number of visits. We tested correspondence between the Council survey and Shmapped data for raw, cleaned, and post-processed (all trips and only those with zero flags) data using Spearman’s rank correlations. We expected to see an increase in correlation as processing progressed and invalid trips were removed from the data.

### Statistical association of trip characteristics with user demographics

We tested whether user-averaged summary characteristics are associated with seven user demographic and personal variables that were collected by the Shmapped app. These are: gender; ethnicity (White or Black, Asian and Minority ethnicities); low vs. high age; low vs. high deprivation (IMD) score for the Lower Super Output Area that the user lives in (as a measure of socioeconomic deprivation); low vs. high time spent outside as a child; low vs. high time spent outside in the past year; and whether the user has access to a private domestic garden. Whilst the two bin categorisation (around the median) is a simplistic approach to attempt to achieve broadly equal samples, it was felt that further subdivision into additional categories would result in sample sizes that were too small and would lack robustness. Limitations of the approach are debated within the discussion section.

Linear models were used to test for associations between these variables and user-averaged summary characteristics: frequency of trips recorded by the user (trips per day), mean duration, mean distance, and mean speed. We also tested for associations with average weekly duration (frequency * duration) and distance (frequency * distance). Only trips with zero flags were used, and only users recording at least 5 trips (with zero flags) were included. The total sample size of users providing full socioeconomic details and recording at least five trips with zero flags was 233. Number of trips, mean duration and mean distance required log-transformation in order to meet model assumptions. F-tests were used to identify significant variables. Statistical analysis was carried out using R [59].

Our study uses a single city as a case study. Sheffield is broadly typical of ex-industrial northern English cities in that it has a higher than average level of socioeconomic deprivation and a high proportion of households comprising semi-detached and terraced housing (Department for Communities and Local Government, 2015). Part of its English industrial heritage is that urban parks were established in the mid-nineteenth century as part of an effort to improve the health of the urban working class (Crompton, 2013), meaning that, in contrast to the UK as a whole, more deprived areas have greater access to urban parks [8,57]. Sheffield is unusual in that it has a large expanse of moorwest immediately to the west of the city, and the city has been branded “the outdoor city” (https://www.theoutdoorcity.co.uk/ accessed on 23/12/2019), both of which may influence residents’ relationship with and attitudes toward urban greenspace and affect their greenspace visiting behaviour.

The reason for the use of a single city is that the app was developed for a project investigating how greenspaces in Sheffield specifically can improve residents’ health and well-being [55]. Moreover, the app (Shmapped–on which this work is based) requires geofences around greenspaces and identification of entrance points. Creation of geofences for Sheffield was facilitated by partnership with the City Council, who shared with us their audit of green and open spaces for leisure and recreation. Ordnance Survey Greenspace data could not be utilised as it was not available when the app was coded (2016). However, the greenspace audit data does not include the extensive areas of countryside that are within easy visiting distance of many of the city’s residents. It should be noted that the creation of a comprehensive access point dataset is a time-consuming task [57]. The single study area also means that we cannot capture more distant, out-of-city visits for Sheffield residents; while only out-of-town trips (i.e. trips to Sheffield) were captured for residents of other places.

## Results

### Trip summary characteristics

**Fig 2:**
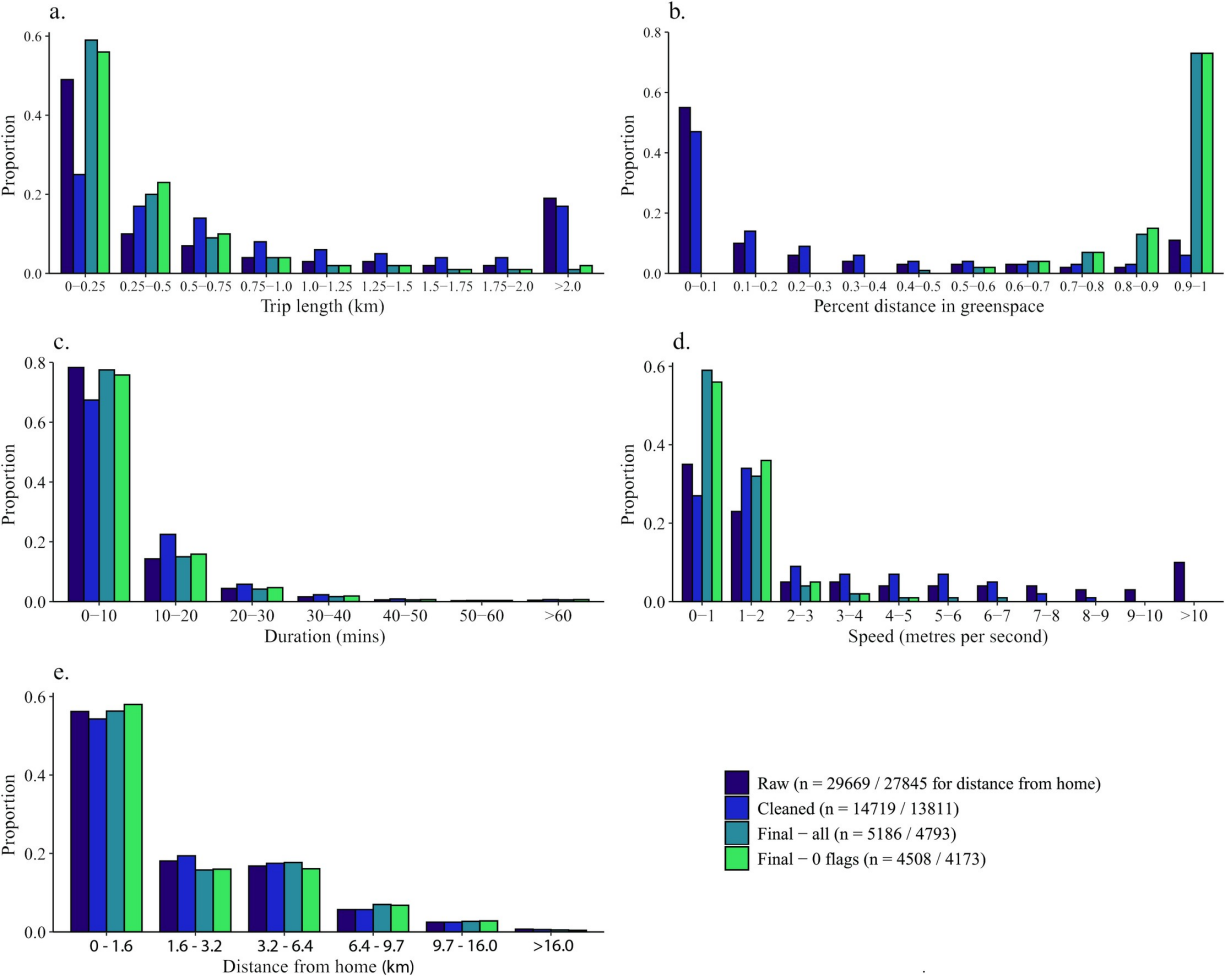
Distribution of trip data characteristics at various stages of processing showing (a) trip length; (b) proportion of the trip in greenspace; (c) trip duration; (d) trip speed; (e) distance from home location. NB distance from home uses imperial units to match intervals from MENE and Sheffield City Council data.

Details of the variables used can be found in Table 3. In total 240 participants from Shmapped generated 29,669 trips that were identified following minimal processing (i.e. splitting only by time gaps, with no cleaning or post-processing). The trips had a median duration of 2 minutes 41 seconds and median distance of 262 m. Of this, a median of 20 m were spent within greenspaces. The median trip-average speed was 1.5 m/s (5.4 km/h). The median distance of trip start from home, for users who gave their home postcode, was 1,310 m. The distribution of these characteristics is shown in Fig 2. All of these characteristics are heavily right-skewed; means and standard deviations are shown in Table 4, although given the skew these should be interpreted with caution. Note that the trip length and speed appear to have large numbers of long and fast trips in Fig 2. This is due to inclusion of trips with unreasonably long length/high speed, likely due to the inclusion of vehicular travel outside of greenspaces but within 10 m of boundaries. These data represent uncleaned data for subsequent comparison.

**Table 3:** Coding of demographic and personal variables tested for associations with trip-level characteristics of visits to greenspace.

Variable	Coding	Notes
Gender	Male (n = 156) / Female (n = 81)	Users of other genders were excluded due to low numbers.
Ethnicity	White (n = 214) / Black, Asian and Minority Ethnic (n = 21)	Black, Asian and Minority ethnicities were aggregated due to low numbers.
Age	Low group: 18–33, n = 126 High group: 34–71, n = 112	Continuous variable split into high/low groups.
Index of Multiple Deprivation	Low group: 19,631–32,816, n = 120 High group: 669–19,414, n = 120	IMD scores are national rank of deprivation, where 1 = most deprived. Continuous variable split into high/low groups. Some data missing due to home postcode not reported.
Time spent outside as a child	Low group: 1–3 (n = 155) High group: 4–5 (n = 83)	Reported by users on a 5-point Likert scale, where 1 = none and 5 = a lot. Variable split into high/low groups.
Time spent outside in the last year	Low group: 1–3 (n = 198) High group: 4–5 (n = 40)	Reported by users on a 5-point Likert scale, where 1 = none and 5 = a lot. Variable split into high/low groups.
Access to a private domestic garden	Yes (n = 51) / No (n = 186)	

**Table 4:** Average characteristics of trips relating to visits to greenspace—pre-processing; following cleaning; and following post-processing (with any number of flags highlighting potentially problematic trip features; 0 or 1 flags; and 0 flags). Characteristics shown as averages across all trips, and as averages of by-user means.

		By trip					By user mean				
		Raw	Cleaned	All trips (max # flags = 2)	Trips with up to 1 flags	Trips with 0 flags	Raw	Cleaned	All trips (max # flags = 2)	Trips with up to 1 flags	Trips with 0 flags
Count		29,669	14,719	5,186	5,141	4,509	888	772	577	576	561
Duration (mins)	Mean	6.5	9.7	7.8	7.9	8.3	5.3	8.8	7.3	7.3	7.7
	Median	2.7	6.8	4.6	4.7	4.9	4.9	7.9	5.9	5.9	6.0
	St. Dev.	10.1	10.6	9.7	9.8	10.2	3.8	5.4	5.8	5.8	6.5
Trip length (m)	Mean	1,311	1,146	362	362	388	1,040	957	341	340	353
	Median	262	621	190	190	212	680	794	256	255	258
	St. Dev.	2,436	1,477	541	541	558	1,114	705	309	309	320
Distance inside greenspaces (m)	Mean	192	202	334	334	361	184	200	311	311	325
	Median	20	71	169	169	194	124	130	231	231	235
	St. Dev.	495	440	509	510	529	222	285	293	294	306
Average speed (metres per second	Mean	3.9	2.4	1.0	1.0	1.0	3.8	2.2	1.1	1.1	1.0
	Median	1.5	1.5	0.8	0.8	0.9	2.9	1.9	0.9	0.9	0.9
	St. Dev.	40.9	2.2	1.1	1.0	0.8	5.9	1.3	0.9	0.8	0.7
Distance from home to start of trip (m)	Mean	2,524	2,564	2,546	2,537	2,457	3,738	3,424	2,903	2,906	2,858
	Median	1,310	1,434	1,286	1,275	1,215	1,692	1,622	1,558	1,559	1,508
	St. Dev.	4,907	4,282	4,149	4,154	3,895	14,301	9,215	6,236	6,241	6,290

During cleaning, 50% of trips were removed, leaving a total of 14,719. The median duration (6 mins 45 secs), trip length (621 m) and distance within greenspaces (71 m) were all increased by cleaning. The median speed was not altered (1.5 m/s or 5.4 km/h), and median distance to home increased only slightly (1,434 m). The skewness of these characteristics was reduced, reflecting the removal of many very short trips, although all remain heavily right-skewed.

**Fig 3:**
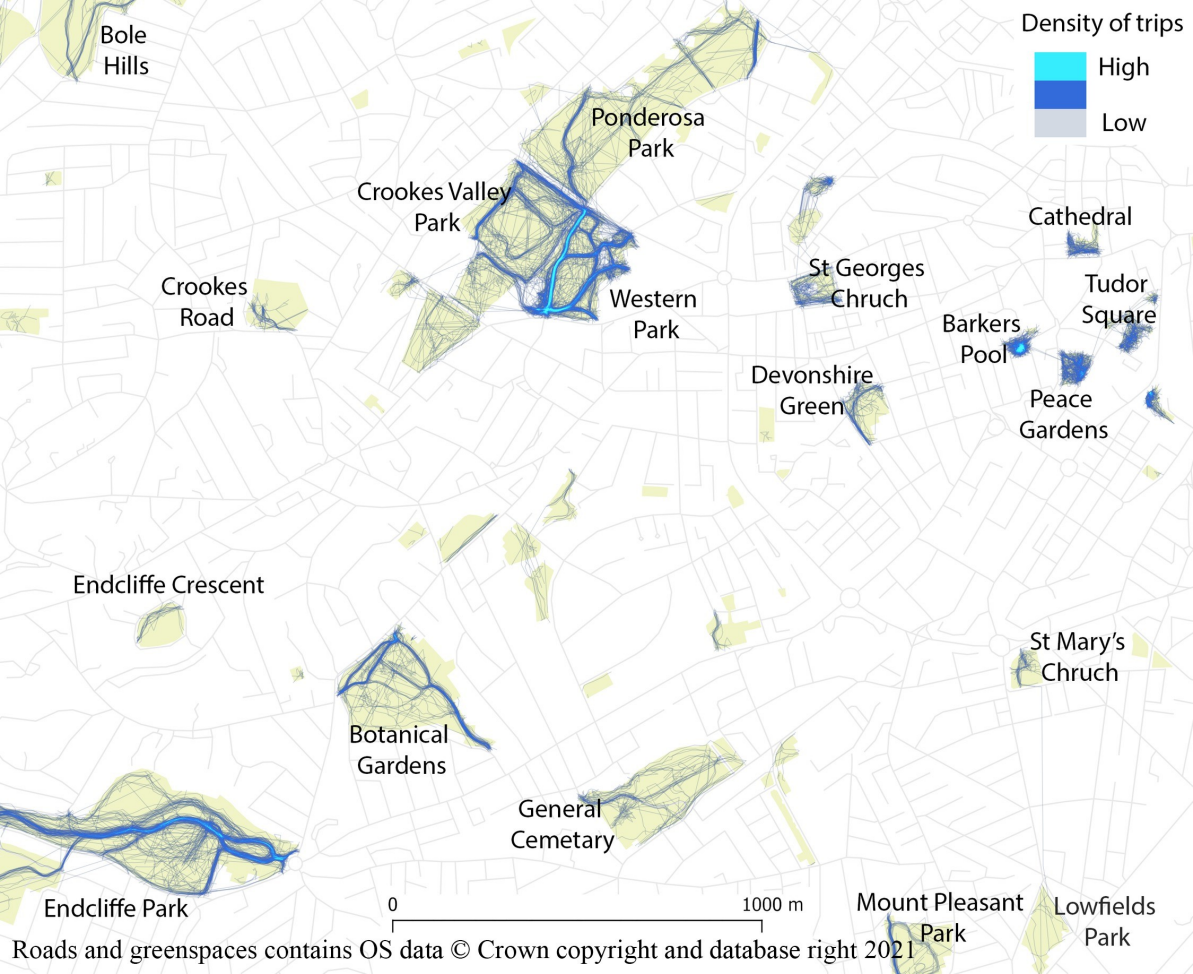
Mapping post-processed trips relating to visits to greenspace.

Following post-processing, 5,186 valid trips were present. The geographic location of the trips are shown in Fig 3. The median duration was intermediate to that of the raw and cleaned data, at 4 mins 36 secs, and the median trip distance was less (190 m), but the distance inside greenspace boundaries increased dramatically to 169 m, reflecting the cropping of trip starts and ends outside greenspaces, and splitting of trips that spent considerable time outside of greenspaces, that was performed during post-processing. The median speed has also reduced to 0.8 m/s (2.9 km/h), due to post-processing efforts to remove trips and parts of trips that were likely undertaken by motorised vehicle. The median distance from users’ homes slightly decreased (1,286 m). The distribution of duration and distance to home are not very different to that of the raw data, while that of trip length and speed are more right-skewed (2). The distribution of percent distance in greenspace is now left-skewed.

Of these 5,186 trips, 399 were flagged due to short distance (<25 m); 41 due to incomplete data at start of end of trip; 84 due to average speed >5 m/s (18 km/h); 113 due to >50% of time or distance spent inside buildings; and 85 due to <50% time and distance spend inside greenspaces. A total of 667 trips have one of these flags, and 45 have two; none have three or more. The number of trips with zero flags is 4,509. Summary characteristics of subsets of trips excluding those with particular flags are shown in S1 Table.

### User-averaged summary characteristics

**Fig 4:**
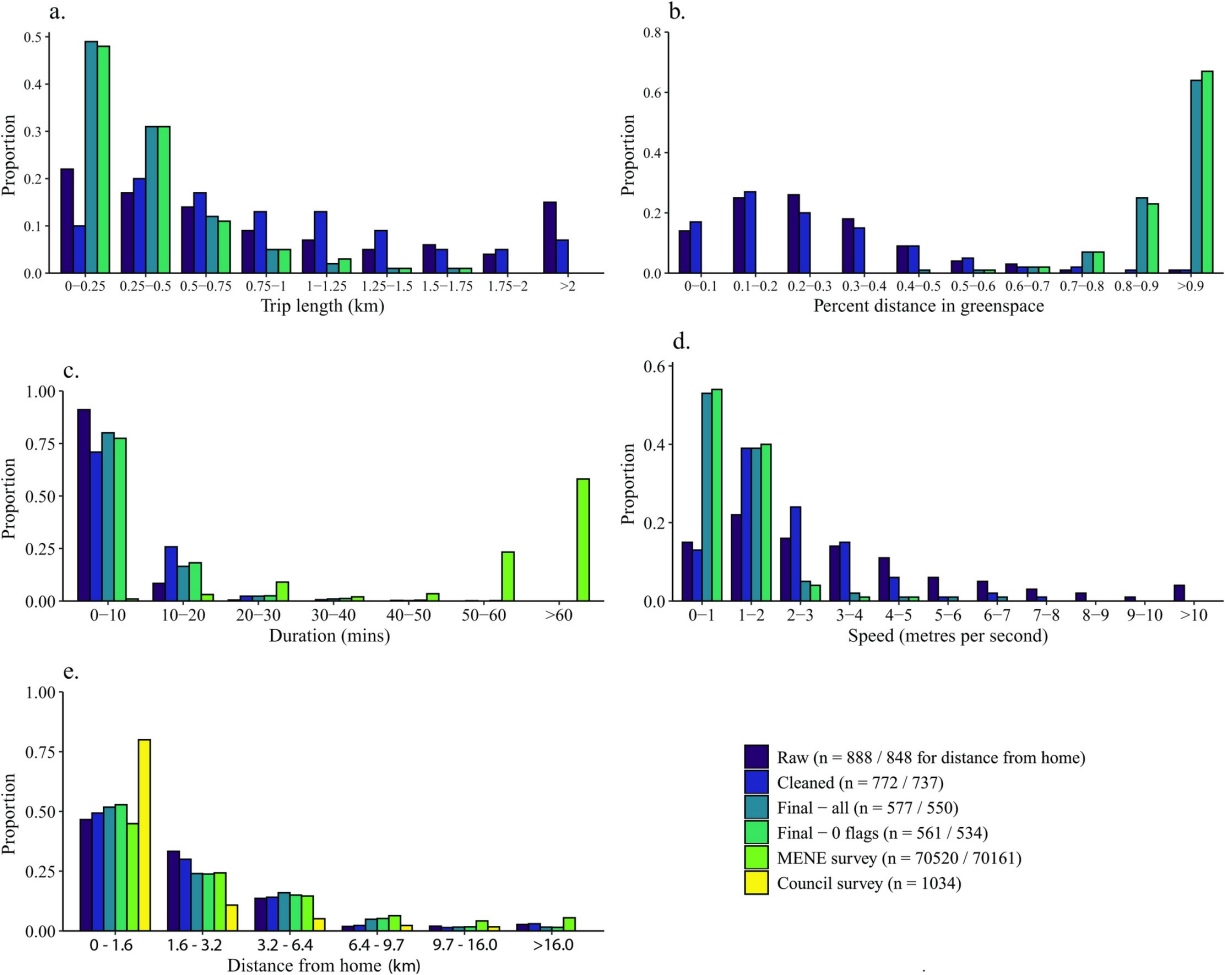
Distribution of trip data characteristics at various stages of processing, averaged by user showing (a) trip length; (b) proportion of the trip in greenspace; (c) trip duration; (d) trip speed; (e) distance from home location. Where available, Monitor of Engagement with the Natural Environment (MENE) (c and e only) and Sheffield City Council survey data (e only) are also shown for comparison. NB distance from home uses imperial units to match intervals from MENE and Sheffield City Council data.

In general, when summary characteristics are taken across user averages, rather than over all trips, all characteristics show higher values (Table 4; distributions shown in Fig 4). This indicates that within individual users there is also right-skew, i.e. many short trips are taken and far fewer short trips. The differences are most profound for the raw data, and are far less following cleaning. This is likely due to cleaning eliminating trips that happen at too great a speed to be non-vehicular travel: although GPS data are only collected within greenspaces, if a user passes multiple greenspaces by car/bus in a short period of time these may still show as single trips in the raw data. Following processing, the differences are smaller again.

Following post-processing, there were 577 users with at least one valid trip. The median user-averaged trip duration and distance (including distance inside greenspaces) are higher than the overall medians, at 5 mins 53 secs and 256 m (231 m in greenspace). The median speed is also slightly higher, at 0.93 m/s (3.3 km/h), and distance from home is further at 1,558 m. There is less right-skew in the distributions of trip length and duration than there is for all trips (compare Fig 4 with Fig 2), indicating that there is right-skewedness within trip distributions for individual users, as well as overall.

There are 15 trips with one flag, and one trip with two, leaving 561 with zero flags. There is less of a difference between user-average medians of trips with zero flags compared to all trips, than there is for non-user-average medians. Summary characteristics for user-averages excluding individual flags are shown in S1 Table.

Summaries of users’ average visit frequency and total duration of trips are shown in Table 5. These show that the mean frequency of visits for all users and including all trips is 1.09 trips per day, with slightly lower values if only users reporting five or more trips and/or only trips with no flags are included. The mean duration is between 7 mins 15 secs for all users/all trips, and 8 mins 18 secs for users with > = 5 trips/trips with zero flags. The total weekly duration of trips is in the range 55 mins 15 secs to 59 mins 0 secs, depending on the combination of users/trips, and the total weekly distance is in the range 2.59 km to 2.75 km. S2 Table contains summaries of the aggregated trip characteristics split by the various demographic and personal variables (including age, gender and ethnicity).

**Table 5:** Average frequency, distance and duration of trips, and total distance and duration extrapolated to daily and weekly totals.

		All users		Users with > = 5 trips	
		All trips	0 flags	All trips	0 flags
N		577	561	269	255
*Summary variables*					
Frequency of trips	Mean	1.09	1.05	1.05	0.99
	SD	0.95	0.89	1.14	1.08
Duration (mins)	Mean	7.26	7.71	8.01	8.31
	SD	5.82	6.50	4.73	4.69
Distance (m)	Mean	341	353	373	383
	SD	309	320	277	270
*Extrapolated duration/distance*					
Duration (mins)	Daily	7.89	8.06	8.43	8.21
	Weekly	55.25	56.42	59.00	57.45
Distance (m)	Daily	371	369	392	378
	Weekly	2598	2586	2745	2644

#### Comparisons with MENE and Sheffield City Council survey data

The distribution of user-averaged trip duration obtained from the MENE survey data is shown alongside that for the Shmapped data in Fig 4C. Whereas the Shmapped data find a right-skewed distribution of duration, the MENE survey finds the opposite, with very few short trips and over half lasting over an hour.

The distribution of user-average distance from home from Shmapped has been compared with data from both the MENE and Sheffield City Council surveys in Fig 4E. The Shmapped data distribution is remarkably similar to that from MENE, although the Council survey finds that more people primarily visit a greenspace within 1 mile of home than is indicated by our data.

### Trip destinations

#### Types of greenspace

**Fig 5:**
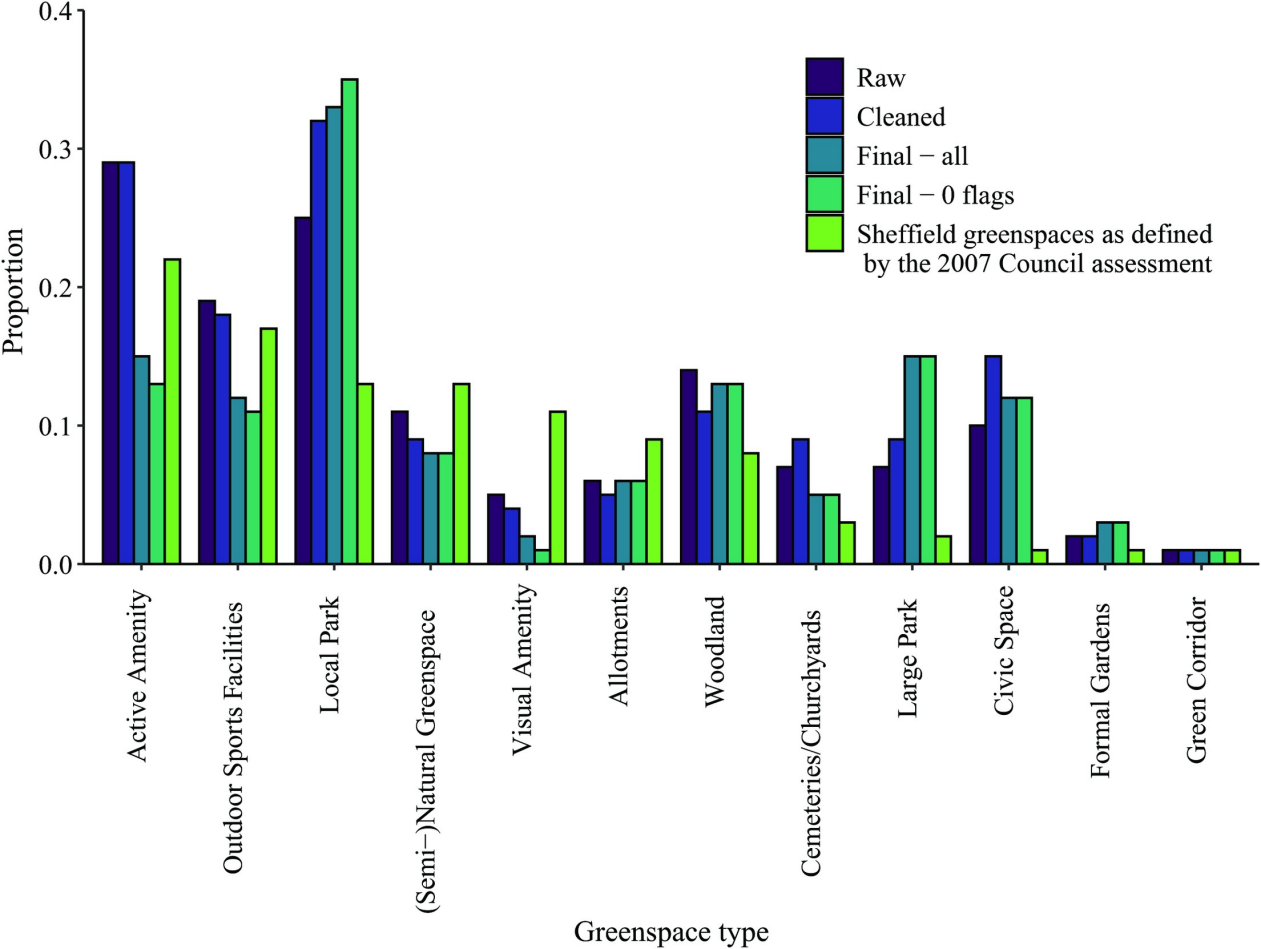
Destinations of trips by greenspace type, at various stages of processing. NB proportions sum more than 1 due to trips visiting multiple greenspaces.

The distribution of types of greenspaces visited is shown in Fig 5. The most common type of greenspace included in the study is active amenity sites (22% of sites are classified as active amenity). While this is the most common type of greenspace visited in the raw and cleaned data, after processing they are substantially under-represented. The same is true of a similar type of greenspace, visual amenity (11%).

The types of greenspace that are most over-represented in the trip data compared to number of sites, especially following processing, are local parks (13%) and large parks (2%). Civic spaces (1%) are also over-represented.

The MENE survey has several categories of greenspace type that can be matched to those used here. The MENE category “park in a town or city” comprises 53% of visits to greenspaces within towns and cities, which is very similar to the 50% of trips in our data that included local and large parks. 7% of MENE trips were to “playing fields or other recreation areas”, compared to 11% of Shmapped trips to outdoor sports facilities. 3% were to “woodland or forest”, compared to 13% in Shmapped; and 1% were to allotments; compared to 1% in Shmapped.

#### Validation against Sheffield City Council survey data

Regarding correspondence between number of users who have visited individual greenspaces, and respondents to the Sheffield City Council survey reporting their most frequently visited greenspace, the correlation between these numbers is increased by data processing (Table 6). When only users who recorded at least 5 trips are considered, the Spearman’s correlation between fully processed trips with zero flags and the Council survey data is 0.40, compared to 0.28 for raw, unprocessed data. When all users are considered, increase in correlation is less, though still present (rho = 0.49 compared to 0.46).

**Table 6:** Spearman’s rank correlation coefficients of number of respondents to Sheffield City Council Parks and Countryside survey who most frequently visit individual greenspaces, with number of Shmapped users who have visited those greenspaces.

	All users	Users with at least 5 trips
Survey vs raw Shmapped data	0.46	0.28
Survey vs cleaned	0.46	0.26
Survey vs final (all)	0.47	0.37
Survey vs final (0 flags)	0.49	0.40

### Association of trip characteristics with demographic factors

#### Average trip duration

Mean trip duration is strongly associated with demographics factors, specifically with ethnicity and age (see Table 7A). Ethnic minority users make trips that are 33% shorter (averaged across the user’s trips) than trips by people of White ethnicity. Trips by people aged 34 and over are 16% longer than those by people under 34. Garden access is approaching significance, with trips by those having access to a garden being 13% shorter. Gender, IMD, and time spend outside either as a child or in the past year do not show significant associations.

**Table 7:** Results of ANOVAs testing relationships between demographic factors and trip characteristics for (a) mean duration; (b) mean distance; (c) mean speed; (d) trip frequency; (e) total duration in greenspace; and (f) total distance in greenspace.

a. Mean duration (mins) of trips (log-transformed) Overall F statistic = 4.439 on 7 and 225 df, p <0.001				
	Sum of Squares	Mean Square	F value	P value
Gender (longer duration = males)	0.24	0.24	1.02	0.31
**Ethnicity (longer duration = white ethnicity users)**	**4.29**	**4.29**	**17.95**	**<0.001**
**Age (longer duration = those aged 34 to 71)**	**1.46**	**1.46**	**6.10**	**0.01**
IMD (longer duration = least deprived)	0.18	0.18	0.74	0.39
Time spent outside as a child (longer duration = those with more time outside as child)	0.00	0.00	0.01	0.90
Time spent outside in the past year (longer duration = those with less time outside in last year)	0.51	0.51	2.13	0.15
Access to a garden (longer duration = those without access to a garden)	0.74	0.74	3.11	0.08
Residuals	53.77	0.24		
b. Mean distance (km) of trips (log-transformed) Overall F statistic = 4.691 on 7 and 225 df, p <0.001				
	Sum of Squares	Mean Square	F value	P value
Gender (longer distance = males)	0.19	0.18	0.50	0.48
**Ethnicity (longer distance = white ethnicity users)**	**6.56**	**6.56**	**17.61**	**<0.001**
**Age (longer distance = those aged 34 to 71)**	**2.42**	**2.42**	**6.51**	**0.01**
**IMD (longer distance = least deprived)**	**2.10**	**2.10**	**5.65**	**0.02**
Time spent outside as a child (longer distance = those with more time outside as child)	0.69	0.69	1.84	0.18
Time spent outside in the past year (longer distance = those with more time outside in last year)	0.10	0.10	0.28	0.60
Access to a garden (longer distance = those without access to a garden)	0.17	0.17	0.45	0.50
Residuals	83.78	0.37		
c. Mean speed (metres per second) of trips Overall F statistic = 2.689 on 7 and 225 df, p = 0.01				
	Sum of Squares	Mean Square	F value	P value
Gender (higher speed = female)	0.02	0.02	0.09	0.76
Ethnicity (higher speed = ethnic minority users)	0.26	0.26	1.35	0.25
Age (higher speed = aged 34 to 71)	0.43	0.43	2.25	0.14
**IMD (higher speed = least deprived)**	**0.86**	**0.86**	**4.52**	**0.03**
Time spent outside as a child (higher speed = those with more time outside as child)	0.53	0.53	2.81	0.10
**Time spent outside in the past year (higher speed = those with more time outside in last year)**	**1.48**	**1.48**	**7.80**	**0.01**
Access to a garden (higher speed = those with access to a garden)	0.00	0.00	0.00	0.98
Residuals	42.58	0.19		
d. Trip frequency (per day) (log-transformed)				
	Sum of Squares	Mean Square	F value	P value
**Gender (more trips = female)**	**4.40**	**4.39**	**6.88**	**0.01**
Ethnicity (more trips = white ethnicity users)	0.48	0.48	0.74	0.39
Age (more trips = aged 34 to 71)	0.83	0.83	1.31	0.25
IMD (more trips = least deprived)	1.47	1.47	2.30	0.13
**Time spent outside as a child (more trips = those with more time outside as child)**	**2.66**	**2.66**	**4.16**	**0.04**
Time spent outside in the past year (more trips = those with less time outside in last year)	0.03	0.03	0.04	0.84
Access to a garden (more trips = those without access to a garden)	0.04	0.04	0.07	0.80
Residuals	143.71	0.64		
e. Total duration in greenspace (mean duration * frequency) (log-transformed)				
	Sum of Squares	Mean Square	F value	P value
**Gender (longer total time = female)**	**6.71**	**6.71**	**6.68**	**0.01**
**Ethnicity (longer total time = white ethnicity users)**	**7.62**	**7.62**	**7.58**	**0.01**
**Age (longer total time = aged 34 to 71)**	**4.50**	**4.50**	**4.48**	**0.04**
IMD (longer total time = least deprived)	2.67	2.67	2.66	0.10
Time spent outside as a child (longer total time = those with more time outside as child)	2.47	2.47	2.46	0.12
Time spent outside in the past year (longer total time = those with less time outside in last year)	0.78	0.78	0.77	0.38
Access to a garden (longer total time = those without access to a garden)	1.14	1.14	1.14	0.29
Residuals	226.15	1.01		
f. Total distance in greenspace (mean distance * frequency) (log-transformed)				
	Sum of Squares	Mean Square	F value	P value
**Gender (longer total distance = female)**	**6.38**	**6.38**	**5.75**	**0.02**
**Ethnicity (longer total distance = white ethnicity users)**	**10.56**	**10.56**	**9.52**	**0.00**
**Age (longer total distance = aged 34 to 71)**	**6.10**	**6.10**	**5.50**	**0.02**
**IMD (longer total distance = least deprived)**	**7.09**	**7.09**	**6.39**	**0.01**
**Time spent outside as a child (longer total distance = those with more time outside as child)**	**6.04**	**6.04**	**5.44**	**0.02**
Time spent outside in the past year (longer total distance = those with more time outside in last year)	0.02	0.02	0.02	0.88
Access to a garden (longer total distance = those without access to a garden)	0.38	0.38	0.34	0.56
Residuals	249.57	1.11		

#### Average trip distance

Mean distance is also highly significantly associated with demographic factors (see Table 7B). Ethnicity and age are again important, with Ethnic minority users covering 33% less distance per trip, and those by people aged 34 or over covering 23% more. IMD is also important, with trips by people living in the less deprived half of LSOAs covering 20% less distance. Gender, access to garden, and time spend outside either as a child or in the past year did not show significant associations.

#### Average trip speed

There are also significant associations between demographic factors and mean speed (see Table 7C). IMD is again significant, with those living in the less deprived half of LSOAs having a 13% higher speed than people living in less deprived LSOAs. Time spent outside in the past year is also significant, with people who have spent more time outside having a 25% faster speed. Gender, age, ethnicity, access to garden, and time spend outside as a child did not show significant associations.

#### Trip frequency

Trip frequency is significantly associated with demographic factors (see Table 7D). Women make 31% more trips than men, and people who spent more time outside as a child make 26% more trips then those who spent less time. Age, ethnicity, IMD, access to garden, and time spend outside in the past year did not show significant associations.

#### Total duration

Total trip duration (i.e. mean trip duration * trip frequency) is also strongly associated with demographic factors (see Table 7E). Women have a total trip duration that is 30% longer than that of men, while that of people aged 34 and over is 31% longer than that of people younger than 34. Ethnic minority users spend 34% less time making trips to greenspace than White users. IMD, access to garden, and time spend outside either as a child or in the past year did not show significant associations.

#### Total distance

There are also associations between demographic factors and total trip distance (mean trip distance * trip frequency) (see Table 7F). Nearly all demographic factors reach significance. Women have a total distance that is 29% longer than that of men; people aged over 34 travel 39% further than those under 34; and Ethnic minority greenspace users travel 34% more distance than White users. People from the less deprived half of LSOAs travel 41% further than those from more deprived LSOAs. Finally, people who spent more time outside as a child travel 40% further than those who spent less. Only time spent outside in the past year and access to a garden are not significant.

S3 Table demonstrates how the results of the relationship with demographic factors change when removing data with least confidence through the data cleaning process.

## Discussion

### Evaluation of processing approach

We used a procedural approach to process the GPS data. The procedural rules were derived from a combination of literature review and trial-and-error investigation of appropriate values for our context. However, it remains possible that our selected rules are not optimal. For example, the period of time used to signify the end of a trip varies from as little as 45 seconds to as many as 900 [39]. One study reported a mean bicycle speed of greater than 5 m/s (18 km/h) [47], which would fall within the range flagged as an uncertainty criterion in this study. Our use of flags partially mitigates these concerns, by making it possible to make a range of inferences with varying levels of certainty in the data.

The alternative to the procedural approach is machine learning, which is increasingly commonly used especially in detection of trip modes [39,44]. Machine learning can achieve high accuracy rates, but it is unclear to what extent the tools developed for one dataset can be applied to other datasets [40]. Implementation of machine learning was beyond the scope of this study, for several reasons: our sample size was relatively small for machine learning methods [44,47]; we do not have accelerometer data, which is typically necessary to distinguish modes with similar speeds [41,44,47]; and we are interested exclusively in non-vehicular travel, which we felt could be distinguished procedurally.

We decided not to incorporate a smoothing step into our procedure. This is in contrast to most studies using GPS data [37–39,45,49], and means that calculated distances may be incorrect due to failure to smooth out signal loss- or scatter-related errors caused by building canyons and tree canopy cover. However, in our case, even small smoothing bandwidths resulted in inaccuracies caused by GPS traces tracking along the outside of greenspaces being bent into greenspaces at corners.

Another issue with our approach is that we have attempted to infer from the data when a trip begins and ends. Users may in fact subjectively experience trip starts and ends differently. For example, users may consider a trip to greenspace to include the approach, once the greenspace is within sight. We have had to choose an arbitrary period of time spent outside of greenspaces to split trips, when in reality a user may experience visits to two greenspaces as part of the same trip even when separated by a longer period, especially if the intervening distance is travelled away from busy roads and/or surrounded by vegetation. Inference was necessary because by design the app did not ask users to identify the starts and ends of the trips; and studies that have used a protocol of asking users to report this information often find a low response rate, due to forgetting or not seeing a personal benefit to providing this data [37,67,68].

Nevertheless, our processing approach is validated by comparison with the results of Sheffield City Council’s survey. Correspondence between the number of users visiting individual greenspaces, and survey respondents reporting sites as their most visited greenspace, is increased incrementally by cleaning, post-processing, and application of flags indicating our level of certainty in the validity of trip data (Table 6).

### Characteristics of trips to greenspace

A major challenge to understanding how to harness public urban greenspace as a tool for improving residents’ health and well-being is the dearth of data available on how people use greenspace [3,5,20]. We have used GPS tracking data collected by a mobile device app to illustrate how residents of an English city use their urban greenspace: (1) how long users spend in greenspaces; (2) how far they travel within them; and (3) how far from home they travel to visit them; (4) average speeds of users; and (5) types of greenspaces visited.

Even after cleaning and post-processing, most of the trips captured by Shmapped are short: 75% are under 10 minutes long and cover less than 500 m (Fig 2A and 2C). This is in stark contrast to the average trip duration of the MENE survey, in which more than 50% of trips last over an hour (Fig 4C). MENE specifically asks respondents about trips *to* greenspace; the contrast suggests that most day-to-day greenspace exposure is incidental, i.e. is not a trip *to* greenspace, but rather *through* it.

Around 40% of greenspace trips take place more than 1.6 km (1 mile) from user’s homes (Fig 2E). It should be noted that our data do not capture trips by Sheffield residents to more distant greenspaces, as only Sheffield greenspaces are geofenced, meaning that the true distribution of distance may be less right-skewed or may be multi-modal. (The few trips more than 16 km (10 miles) from home are mostly by people visiting from outside of Sheffield.) While we do not have data on either whether trips began at home (instead of e.g. from workplaces), the distribution of user-averaged distance from home is remarkably similar to the MENE survey distribution of distance from trip origin (which may or may not be the respondent’s home) to greenspace (Fig 4E).

The typical movement speed is low, with over half of trips having a total average speed of less than 1 m/s (3.6 km/h) (Fig 2). Typical walking speeds for healthy men and women aged under 70 are around 1.3–1.4 m/s (4.7–5.0 km/h) [69]. A study of walking speeds from Drents-Friese Wold National Park in the Netherlands found that walking speed varied with trip motive [70]. Walkers whose purpose was “social and activities”, “social and relaxation” or “nature and rest” walked more slowly than the average speed found in this study, ranging between 0.7–0.9 m/s (2.5–3.2 km/h), while those engaging in “walking as exercise” were faster, at 1.1 m/s (4.0 km/h) [70]. This suggests that most of the Shmapped users were walking for social, nature-observation and relaxation purposes, rather than exercise, and may have been slowing or stopping to observe their surroundings, to rest and/or to socialise. Given the small number of users travelling as speeds above 2 m/s (7.2 km/h), there appear to have been few cyclists or runners recording trips using Shmapped. Runners typically move at between 2–4 m/s (7.2–14.4 km/h), while most cyclists move between 2–5 m/s (7.2–18.0 km/h) [41,44,46–48].

Parks, both local and large, are shown to be particularly popular destinations for greenspace visits (Fig 5). While these comprise only 15% of the geofenced greenspaces, they are visited on half of all trips. Parks are also popular destinations for respondents to the MENE survey. This is unsurprising given that the urban parks in particular are recognised to fulfil a range of important social, aesthetic, well-being and recreational roles [22,71,72]. Civic spaces are also visited more frequently than would be expected, which is likely because of their city centre location and utility as resting and meeting places. Whilst our data do not explicitly question the purpose of the greenspace visit, the numerous occurrences of singular direct routes within the spaces hints at greenspace usage within wider travel (passing through–for example on the way to work) rather than as explicit destinations themselves.

In contrast, visual and active amenity sites are under-represented in proportion of visits. These are typically small areas of greenspace provided as local greens in residential areas. They are not underrepresented in the raw data, as this may be because the proximity of amenity greenspaces to roads means that many apparent trips in the unprocessed data are undertaken by motorised vehicles. Additionally, some are so small that it takes less than 70 seconds (the minimum time for a trip to be considered valid) to walk through them.

Assuming that the GPS data recorded by Shmapped reflect typical patterns of greenspace usage for the users, the average weekly time spent visiting greenspaces is slightly less than an hour, and the total distance travelled is a little over 2.5 km (Table 5). An hour is 40% of the 150 weekly minutes of moderate exercise recommended by the UK National Health Service (https://www.nhs.uk/live-well/exercise/, date accessed 24/12/2019), although given the low average walking speed it is likely that some users were not walking fast enough for their time spent in greenspaces to count as moderate exercise. An hour is also around 40% of the average weekly time spent walking as found by the Health Survey for England 2012 [73]. Although the relationship between greenspace exposure and health benefits does not plateau until considerably beyond an hour, some studies indicate that an hour is adequate to obtain mental health benefits [74,75]. Another study, however, found that 120 minutes is necessary to provide consistent improvements to health and well-being of a magnitude similar to, for example, living in a low- compared to a high-deprivation area or achieving recommended levels of physical activity [76]. However, given the interaction between nature contact and an individual’s nature connectedness, shorter durations could well provide greater wellbeing benefits for more nature-connected individuals [14].

### Determinants of greenspace-visiting behaviour

Data on how different people use greenspaces are important because greenspace use is known to be influenced by factors including age, gender, ethnicity and deprivation [24–27]. We have investigated the influence of several of these factors on greenspace visitation.

Black, Asian and minority ethnicity is associated with less time spent and less distance travelled in greenspaces, both per trip and overall. This may have health impacts: for example, for pregnant White women in Bradford, UK, birth weight was associated with residential greenness while for Pakistani women it was not, which is likely to be due to differences in greenspace usage [77]. A meta-analysis of studies from North American found that people of White ethnicity were less constrained from visiting parks by cost, transportation, health and knowledge about parks [24]. In the UK, ethnic minorities may obtain different health benefits from the greenspace environment than White people. General health amongst ethnic minorities (excluding people of Indian ethnicity) was more strongly predicted by greenspace use and perceptions than amongst White British or Indian people [28]. This may be related to confounding of ethnicity and deprivation; greenspace has been found to have greater benefits to health amongst deprived groups [6–9]. Furthermore, visiting greenspace with another person was only a predictor of general health for ethnic minorities, reflecting other results that suggest that ethnic minorities have different, more socially-oriented requirements of greenspace visits than White people [27,28].

Age is also associated with time spent and distance travelled in greenspaces, with the older half of people in our study (aged 34–71) spending longer and travelling further than the younger half (aged 18–33). Age is associated with different constraints on greenspace visitation. Younger people tend to be constrained more by time and cost, as well as knowledge of local greenspaces [24]. In contrast, older people are often more constrained by health, availability of someone to go with, safety concerns and availability of transportation [24]. In our study, the younger members of the ‘older’ group are still relatively young (minimum age 34) and therefore less likely to be limited by health concerns. Furthermore, the ‘younger’ group in our study includes many university students, a group who are particularly likely to have time constraints [78]. Age was found not to be significantly associated with speed. This may be due to the modest sample sizes or the categorisation/data binning of the variables (as discussed in more detail within the limitations section).

Our data has showed that deprivation is associated with distance but not duration or frequency; correspondingly, it is also associated with average speed. People from more deprived areas move more slowly and cover less distance than those from less deprived areas. The reduced speed associated with lower physical activity levels of people from deprived areas (through lower levels of running or cycling) may in turn decrease average distances. Lower walking speeds may arise because people from deprived areas tend to have poorer health and lower levels of physical activity [79,80]. Interestingly, in contrast to our finding of no relationship between deprivation and duration or frequency, a study from Birmingham, UK found that people from more deprived areas spent less time walking outside, while one from the US found *higher* duration of walking for transport amongst people from more deprived areas [81,82]. It is possible that our finding arises from the fact that deprived areas in Sheffield have particularly good accessibility to greenspaces, which offsets deprivation-related constraints [24,57].

Time spent outside in the past year is also associated with average speed. This relationship may be due to a relationship between walking speed and general mobility, e.g. ability to cross streets and navigate the local environment, which in turn limits ability to visit even nearby destinations [69,83,84].

Gender is not associated with per-trip characteristics, but women make more frequent trips, and therefore have a greater total distance and duration. This is surprising given that studies generally find women to be more affected by all types of constraints on greenspace visitation, from perceptions of safety and availability of someone to go with, to time and cost, especially relating to family responsibilities [24–26]. The explanation for the observed association may be related to the likelihood of using different modes of transport: Department of Transport data suggest that men may be more likely to travel by car, while women may be more likely to walk [85].

More time spent outside as a child is associated with greater trip frequency and total distance, although not with total duration. This is unsurprising given the important influence childhood habits and attitudes towards greenspace in adulthood [78,86–88]. For example, a survey of English and Scottish adults found that the frequency of visits to greenspace as a child strongly influenced the frequency of visits as an adult [87].

Access to a garden is not associated with any trip characteristics. This supports the finding that people who lack a private garden do not compensate with more frequent visits to public greenspaces [89]. Private gardens have different functions and meaning to public greenspace: they are spaces that simultaneously provide privacy and freedom, and the possibility to create an outdoor space that meets one’s individual requirements e.g. through gardening and installation of facilities [90]. The lack of significant associations in our study is likely due to the non-substitutability of public and private greenspace.

### Limitations

A limitation is that, although marketing of the app (Shmapped) was targeted to capture a representative sample of Sheffield’s population, users were self-selecting [54,55]. Consequently, there are socioeconomic differences between the sample with at least one valid trip and the wider Sheffield population (S1 Fig). 65% of app users are male, whereas only 49% of Sheffield’s population of over-18s are. App users are mostly between the ages of 29 and 40, while Sheffield’s population has a far larger proportion of older people. The area deprivation of areas where app users live was considerably less, on average, than that of Sheffield’s wider population. However, the proportion of users belonging to different ethnic groups was relatively similar to that of Sheffield’s population, with the exception that not many people of Black and Black British origin used the app. This has consequences for the generalisability of results, due to the different greenspace usage behaviour observed both in this study and elsewhere [22–27].

The authors acknowledge that using data extracted from Shmapped, we were unable to incorporate additional socio-demographic variables of interest such as profession. Therefore, it should be acknowledged that associations found could be the result of third variable linkages. Future research is needed to expand on the current exploratory outputs. Due to the relatively modest sample sizes, analysis was undertaken by splitting each variable into two bins with broadly equal sample sizes. Future research should seek larger samples in order to exploit the continuous nature of the majority of socio-demographic variables using more sophisticated analysis such as Generalised Linear Modelling. Additionally, the app was advertised as a mental well-being self-help tool, but it is possible that people who spend more time in outdoor spaces were more likely to download the app and to engage with it throughout the intervention period. The medium of a mobile device app is also more likely to attract particular demographics, namely those who are younger and more technology-friendly [91], as well as those able to afford modern smartphones, which likely explains the age distribution of app users in this study. We also cannot be certain if the trips measured by a smartphone app are representative of all trips to greenspace. There might be certain types of visit where participants were less likely to carry their mobile phones (for example a short trip to walk the dog) which may systematically bias output.

Regarding our analysis of associations between demographic factors and greenspace visiting behaviour, while we have included a variety of demographic factors, there may be other factors influencing greenspace visitation. These include personal factors such as health, education, and responsibilities e.g. to family that limit available time [24–26]; and also wider factors such as inability to get to parks, perceptions of safety and social inclusiveness of the local area [23,25,26]. We have also not in this study been able to analyse park-level influencing factors, such as park quality, which is known to affect both use and health benefits [1,92].

Finally, although we have been able to analyse *where* people choose to visit, we have not been able to investigate *why*, or what they did during their visit (e.g. the extent to which they engaged with nature). This limits our ability to understand how individuals’ past and present experiences influence greenspace visiting behaviour, and to understand the subjective value of visiting greenspaces [35,93].

## Conclusions

In recent years, use of GPS to inform both travel surveys and health research has increased enormously [34–37,39,41,42]. GPS data has the potential to resolve the Unknown Geographic Context Problem by facilitating a detailed understanding of how people experience their local environment on a day-to-day basis [32–35]. It therefore shows great promise as a provider of data on greenspace visitation, rather than simply what greenspace exists, and thereby contributing to the epidemiological understanding of how urban greenspace contributes to the health and well-being of residents [3,5,20].

We have demonstrated a method for processing raw GPS data into useful information about individual trips to greenspaces. Key findings are that the median trip length is 190 m and the median duration is 4 minutes 36 seconds, and that the average user of our app makes just over one trip per day to a greenspace, with a weekly total duration of nearly an hour and total distance of around 2.5 km. However, these statistics are influenced by demographic factors including age (older participants spent more time and covered more distance in greenspaces) and gender (women make more frequent trips to greenspace). Importantly, ethnicity and deprivation also play a role, with ethnic minorities and people from more deprived areas making shorter visits to greenspaces. However, time spent outside as a child seems to positively influence the frequency of trips made as an adult, suggesting that behaviours learnt as a child continue into adult life. While relating specifically to the case study city of Sheffield, these insights suggest a way forward to understanding how greenspace use benefits the various demographics of a city’s population.

## Supporting information

S1 Fig Socioeconomic characteristics of app users compared with Sheffield’s wider population. (DOCX)

S1 Table Average characteristics of subsets of trips excluding flags highlighting trips with potentially problematic features. Characteristics shown both averaged across all trips, and as average of by-user means.
(DOCX)

S2 Table Average characteristics of trip duration, length and speed split by (a) gender; (b) age; (c) ethnicity; (d) deprivation; (e) time spent outside as a child; (f) time spent outside last year; and (g) access to a garden.
(XLSX)

S3 Table Results of ANOVAs testing relationships between demographic factors and trip characteristics–exploring effects when trips with more flags are included. (XLSX)
