# Supplementary material for: Mapping urban greenspace use from mobile phone GPS data
Source: PLoS One. 2021 Jul 7;16(7):e0248622. doi: 10.1371/journal.pone.0248622 (PMC8262795; doi:10.1371/journal.pone.0248622)

# Figure S1. Socioeconomic characteristics of app users compared with Sheffield’s wider population in terms of (a) gender; (b) age; (c) ethnicity and (d) deprivation (Index of Multiple Deprivation - IMD). In (d), decile 1 = most deprived.


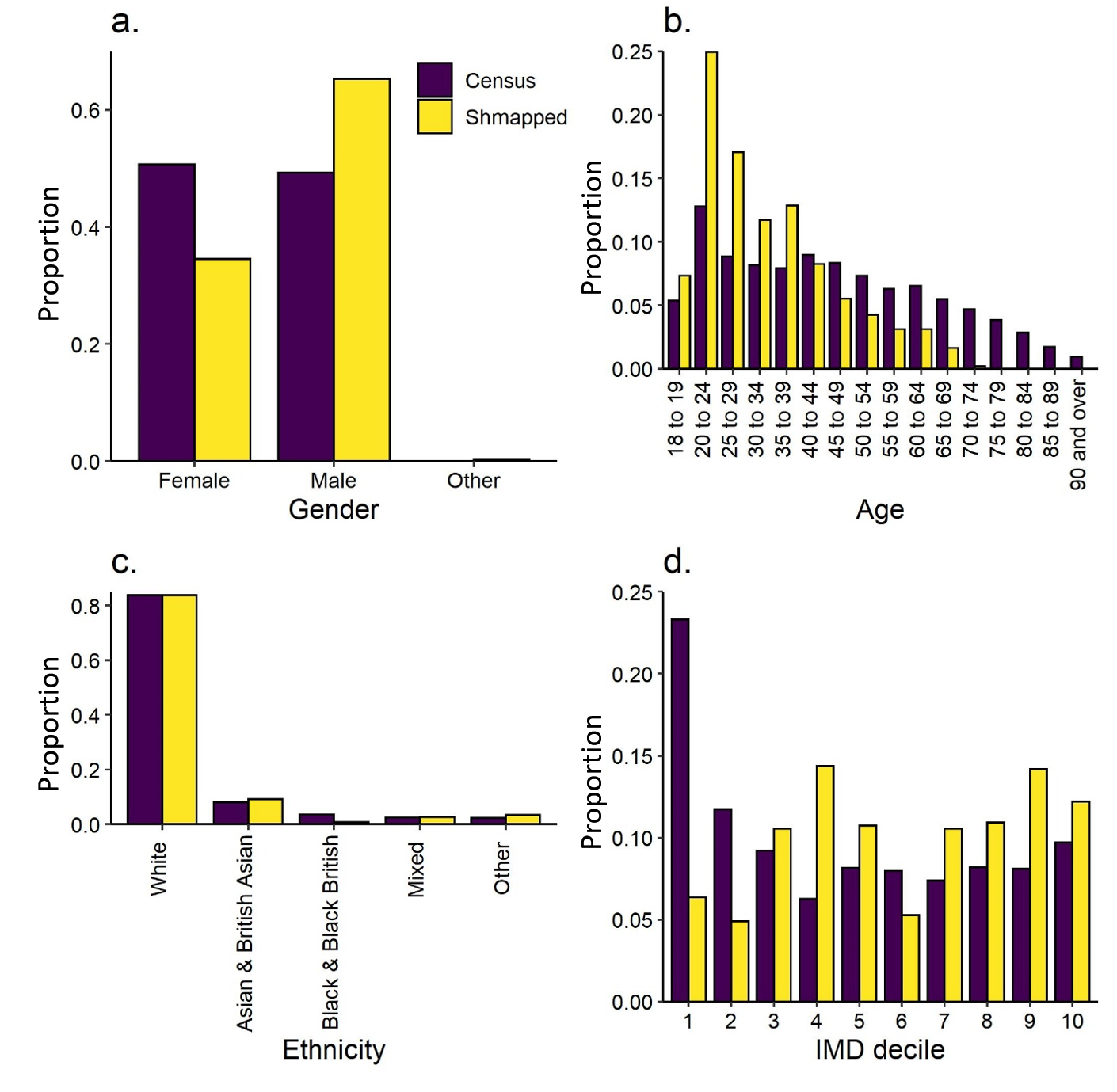

Supplement: S1 Fig — (DOCX) [file pone.0248622.s001.docx]
