# Supplementary material for: Mapping urban greenspace use from mobile phone GPS data
Source: PLoS One. 2021 Jul 7;16(7):e0248622. doi: 10.1371/journal.pone.0248622 (PMC8262795; doi:10.1371/journal.pone.0248622)
Supplement: S1 Table — Characteristics shown both averaged across all trips, and as average of by-user means. (DOCX) [file pone.0248622.s002.docx]

# Table S2. Average characteristics of subsets of trips excluding flags highlighting trips with potentially problematic features. Characteristics shown both averaged across all trips, and as average of by-user means.

|  |  | By trip | | | | | By user mean | | | | |
| --- | --- | --- | --- | --- | --- | --- | --- | --- | --- | --- | --- |
|  |  | Excl. trips with length <25m | Excl. trips with incomplete data | Excl. trips with speed >5mps | Excl. trips >50% in buildings | Excl. trips >50% outside greenspace | Excl. trips with length <25m | Excl. trips with incomplete data | Excl. trips with speed >5mps | Excl. trips >50% in buildings | Excl. trips >50% outside greenspace |
| Count |  | 4787 | 5145 | 5102 | 5073 | 5101 | 571 | 576 | 572 | 576 | 574 |
| Duration (mins) | Mean | 8.1 | 7.9 | 7.9 | 7.8 | 7.9 | 7.5 | 7.3 | 7.4 | 7.3 | 7.3 |
|  | Median | 4.8 | 4.6 | 4.7 | 4.5 | 4.7 | 6.0 | 5.9 | 5.9 | 5.8 | 5.9 |
|  | St. Dev. | 10.0 | 9.8 | 9.8 | 9.8 | 9.8 | 6.3 | 5.8 | 5.9 | 5.9 | 5.8 |
| Trip length (m) | Mean | 391 | 359 | 353 | 367 | 365 | 359 | 339 | 331 | 345 | 342 |
|  | Median | 213 | 188 | 185 | 193 | 191 | 268 | 251 | 246 | 257 | 255 |
|  | St. Dev. | 553 | 540 | 538 | 545 | 544 | 314 | 309 | 306 | 318 | 310 |
| Distance inside greenspaces (m) | Mean | 361 | 332 | 327 | 339 | 338 | 328 | 309 | 304 | 315 | 314 |
|  | Median | 193 | 168 | 165 | 171 | 172 | 237 | 227 | 221 | 233 | 232 |
|  | St. Dev. | 521 | 509 | 509 | 514 | 513 | 297 | 293 | 292 | 301 | 294 |
| Average speed (meters per second | Mean | 1.1 | 1.0 | 0.9 | 1.0 | 1.0 | 1.1 | 1.1 | 1.0 | 1.1 | 1.1 |
|  | Median | 0.9 | 0.8 | 0.7 | 0.8 | 0.8 | 1.0 | 0.9 | 0.9 | 0.9 | 0.9 |
|  | St. Dev. | 1.1 | 1.0 | 0.8 | 1.1 | 1.1 | 0.9 | 0.9 | 0.7 | 0.9 | 0.8 |
| Distance from home to start of trip (m) | Mean | 2505 | 2537 | 2529 | 2526 | 2543 | 2922 | 2874 | 2893 | 2890 | 2902 |
|  | Median | 1265 | 1276 | 1263 | 1265 | 1285 | 1557 | 1555 | 1546 | 1560 | 1559 |
|  | St. Dev. | 3869 | 4153 | 4170 | 4157 | 4159 | 6267 | 6210 | 6265 | 6243 | 6250 |
